# Cutaneous Malignancies in Tattoos, a Case Series of Six Patients

**DOI:** 10.3390/curroncol28060398

**Published:** 2021-11-15

**Authors:** Marike Leijs, Hannah Schaefer, Albert Rübben, Claudio Cacchi, Thomas Rustemeyer, Sebastiaan van der Bent

**Affiliations:** 1 Department of Dermatology, St. Nikolaus Hospital, 4700 Eupen, Belgium; Albert.Ruebben@post.rwth-aachen.de; 2 Department of Dermatology and Allergology, RWTH Aachen University, 52074 Aachen, Germany; hannah.schaefer@rwth-aachen.de; 3 Institute of Pathology, RWTH Aachen University, 52074 Aachen, Germany; ccacchi@ukaachen.de; 4 Academic Tattoo Clinic Amsterdam, Department of Dermatology and Allergology, Amsterdam UMC, 1081 HV Amsterdam, The Netherlands; t.rustemeyer@amsterdamumc.nl; 5 Tattoo Clinic (Tattoo Poli), Department of Dermatology, Alrijne Ziekenhuis Leiden, 2334 CK Leiden, The Netherlands; sasvanderbent@alrijne.nl

**Keywords:** tattoo, tattoo ink, melanoma, basal cell carcinoma, squamous cell carcinoma, polyaromatic hydrocarbons, tumor promotion, endocrine disruptors

## Abstract

Background: A variety of side effects following the tattooing of the skin were reported over the years. Analytical studies showed that some tattoo inks contain harmful compounds. Methods: We presented six patient cases with cutaneous malignancies in tattooed skin and performed an extensive literature research. Results: Two patients with black ink tattoos that were diagnosed with malignant melanoma raises the number of described cases to 36 patients. One of the patients developed an immunologic reaction limited to the tattoo area after treatment with a targeted immune therapy. In the other patient, the malignancy (malignant melanoma) was fatal. Basal cell carcinoma was seen in four patients with tattoos containing varying ink colors (black, green, red). This increased the number of described patient cases to 18. Although some ink components and their cleavage products have carcinogenic properties, epidemiological evidence for a causative correlation fails. Further epidemiologic studies on tattoos and malignancies, as well as on the appearance of naevi in tattoos, are necessary. Determining the type of mutation might be helpful to separate sun-induced tumors from skin cancers due to other pathogenic mechanisms.

## 1. Introduction 

Tattooing is defined as the artificial introduction of exogenous pigments into the dermis [1]. Both the incidence of tattoos and malignant melanoma have shown an extreme increase in recent decades [2,3,4,5]. The occurrence of tattoos in industrial countries reaches up to 36% of the German population, and 24–46% of the population of the USA are tattooed [3,6]. Especially, young respondents were more likely to have even multiple tattoos [3,5,7]. 

Meanwhile, several adverse events are reported on tattooed skin [7,8]. These include not only type IV allergic reactions (allergic contact dermatitis), but also skin cancer [7,9,10]. Other non-malignant adverse events include infections, scars, keloid, blow-out and the occurrence of auto-immune skin diseases such as sarcoidosis and psoriasis [11,12,13]. 

The complications of infectious origin include bacterial infections of the skin (most common: staphylococcus, streptococcus, Pseudomonas aeruginosa and Escherichia coli) and viral infections in the formation of various skin lesions (human papilloma virus, pox virus, herpes virus). In addition, serious infectious diseases such as the human immunodeficiency virus (HIV) or hepatitis virus (Hep. B/C) might be transmitted during tattooing [12]. 

Since 1938 [5,14], several benign and malignant skin lesions occurring in tattoos have been reported [10,14,15,16,17,18]. Malignant lesions of the skin reported in tattooed areas included malignant melanoma, basal cell carcinoma, squamous cell carcinoma, dermatofibrosarcoma protuberans, lymphoma and keratoacanthoma [19]. A total of 36 cases (32 male and 3 female, 1 NA) of malignant melanoma arising in tattoos were reported (Table 1). 

Until today, the link between the clinical appearance of malignancies in tattoos and the containment of toxic compounds was not proven. Most tattoo inks consist not only of insoluble pigments but also additives, dispersants and preservatives. The toxicological risk of some of the used compounds is relatively unknown [81]. The predominantly used ink color is black (carbon black), often containing compounds such as potential genotoxic polyaromatic hydrocarbons (PAHs) [82]. Although today’s ink compounds are assumed to contain less genotoxic compounds, they still consist mainly of pigments that were developed for other purposes (e.g., plastics, printing, textiles, cosmetics and even automotive coatings) and safety assessments/risk assessment are lacking [83]. Nevertheless, heavy metals still have a large proportion of tattoo ink [81]. An analysis of commercially used inks revealed that the most used colorants were titanium, barium, aluminum and copper [84,85]. However, it became clear that more harmful compounds such as antimony, arsenic, cadmium, chromium, cobalt, lead, and nickel were mostly present as contaminants [81,84,85]. The amounts of metals in the composition of tattoo inks seems highly variable between brands and colors, even in pigments with the same base color [86].

Eventually, about 10% of all tattooed individuals choose to remove their tattoo, mostly by using laser-assisted tattoo removal where the cleavage of tattoo ink occurs in largely unclear products. Even without laser-assisted removal, tattoos fade in time [87]. Pigments are most likely to be metabolized by enzymes such as azo reductases. Through such a pathway, the colorants might split in aromatic amines [87].

Due to the limited number of published cases of tumors in tattoos, there is insufficient epidemiological evidence for a correlation with (possible) harmful compounds in tattoo ink. With six case series and an overview of the literature, we increase the number of published cases and aim to broaden the pathophysiological knowledge on malignancies in tattoos and the possible role of tattoo ink.

## 2. Materials and Methods 

### 2.1. Study Cohort 

Retrospectively, we included patients, who developed a malignancy in their tattoo, from the Department of Dermatology and Allergology at the RWTH Aachen University in Aachen, Germany (2005–2020); the Department of Dermatology and Allergology, UMC Amsterdam in Amsterdam, The Netherlands (2015–2020), and the Department of Dermatology, Alrijne Ziekenhuis Leiden, The Netherlands (2021). A total of 6 patients (6 males) were included. Informed consent for the usage of photos was obtained for 5 of the patients. For one patient with malignant melanoma who died, no informed consent was possible since the contact details of the relatives were not valid anymore, so no clinical pictures of this patient were shown.

### 2.2. Literature Research and Evaluation of the Data

The literature study was performed in PubMed and Google Scholar with the following search terms: “tattoo(ing)”/“tattoo” and “ink” in combination with “melanoma”, “malignant melanoma”, “basal cell carcinoma”, “squamous cell carcinoma”, “keratoacanthoma”, “dermatofibrosarcoma protuberans” and “cutaneous leiomyosarcoma”, “cancer”, “allergy”, “complication”, “dyes”, “heavy metals”, “infection”, “phototoxicity”, “pigments”, “preservatives”, and “toxicology”. The databases “PubChem” and “European Chemicals Agency” (ECHA) were used to gather information about the toxicity of the tattoo ink ingredients. The database on current cancer statistics of the Robert Koch Institute (RKI) was used to compare the characteristics of the published case studies with the general population in Germany. No further criteria were used to select the sources. 

In our overview of the literature (Table 1), we present all the papers published up to this point with cases concerning malignancies in tattoos. Because of the scarce amount of papers published on this topic, apart from the correlation between the occurrence of malignant skin lesions in tattooed skin regions, there were no other inclusion criteria for the selection of the literature. The results of the literature search were summarized in tabular form. Details can be found in the Prisma flow diagram (Figure S1).

## 3. Results

We present six male subjects who developed malignancies in tattooed areas of the skin.

### 3.1. Cases with the Formation of Melanoma in Tattooed Skin

**Figure 1:**
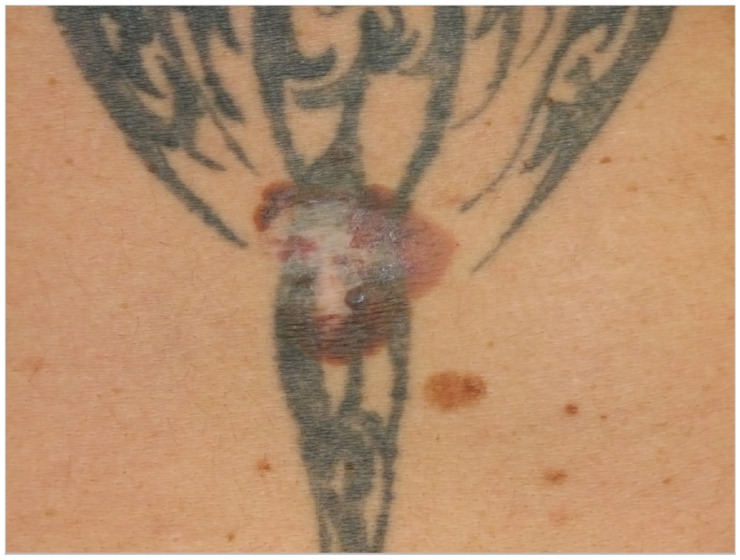
A recurrent skin lesion on the back which was previously removed 18 years ago.

Patient 1: A 52-year-old man (smoker) presented to our clinic for a skin cancer screening in 2018. Although during the examination a suspicious skin lesion at the lower back was found, no other malignancies were found during an extensive skin examination. The patient reported that a skin lesion was already removed 18 years ago but re-appeared since. The patient reported no history of trauma. The examination revealed that the suspicious skin lesion was a 4 × 6 cm large, irregularly pigmented, partly semi-circular maculae in the area of a tattoo (see Figure 1). We suspected a malignant melanoma and performed an excision of the lesion in toto. Histopathology revealed a superficially spreading malignant melanoma (SSMM) with a thickness of 0.7 mm (pT1a, N1a, M0, R0). Sequencing did not reveal pathogenic mutations in genes BRAF, NRAS or KIT. Subsequently, the SSMM was re-excised with 1 cm margins and, because of the size of the melanoma in combination with histopathological signs of regression, we decided to perform a sentinel lymph-node excision. There was a positive sentinel lymph node in the right groin containing micro metastasis up to 1.2 mm, so the patient was diagnosed with stage IIIA (AJCC 2017) malignant melanoma. Subsequently, a right inguinal lymph node dissection was performed (0/12) and an adjuvant immuno-therapy with Nivolumab (3 mg/kg every 2 weeks) was given for 12 months. The patient was introduced to us again 5 months later with cutaneous side effects (toxicity grade 1) on the abdomen induced by the targeted immunotherapy with nivolumab. We examined palpable small papules in the area of the tattooed skin with emphasis on the black color. We suspected an immunological reaction in this area triggered by the black color ink, most likely a granulomatous skin reaction as seen in another study [88]. The patient presented himself to regular follow-up examinations and has had no recurrence of the SSMM to date. 

**Figure 2:**
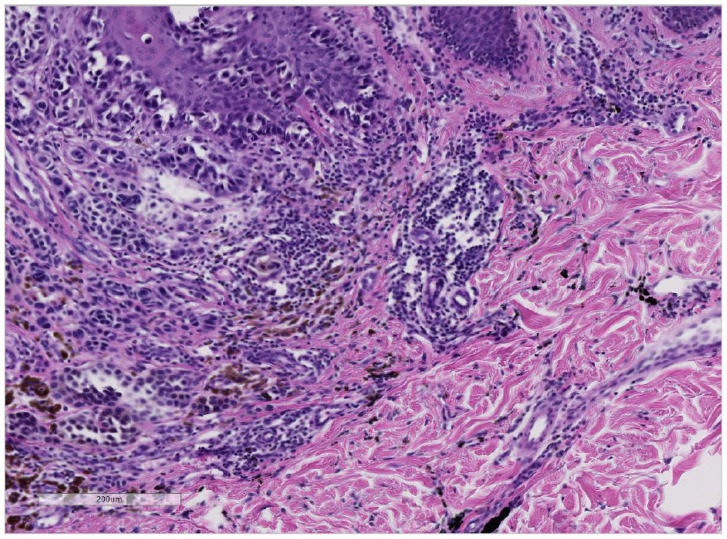
(Hematoxylin and Eosin 10×): Patient No. 2: the histology shows malignant melanocytes with atypia in the epidermis and in the dermis. Note the difference of color between melanin pigment (brown) and the black tattoo pigment (right/bottom).

**Figure 3:**
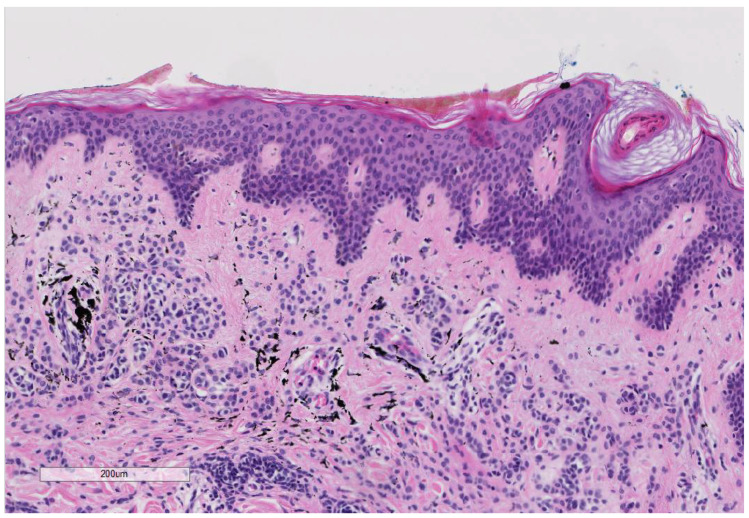
(Hematoxylin and Eosin 10×): A benign melanocytic nevus within the tattoo of patient No. 2: The slide shows benign small melanocytes without atypia in the in the dermis in association with the fine-granular black tattoo pigment.

**Figure 4:**
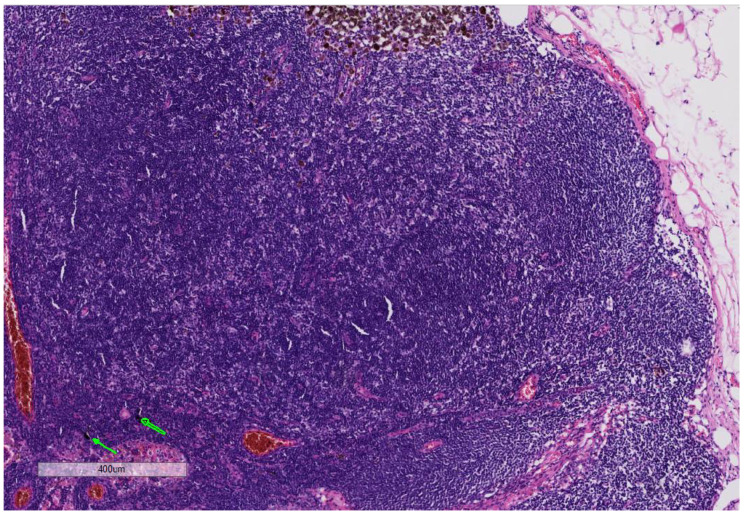
(Hematoxylin and Eosin 5×): Patient No 2: The slide demonstrates melanoma cells in the lymph node with brown melanin pigment and black ink in the same lymph node (arrows).

Patient 2: The second case (33-year-old male) presented himself with a melanoma on the left shoulder blade/scapula (tumor thickness 6.0 mm, Clark-level IV, pT4a). No other malignancies were found during an extensive skin examination. Anamnesis revealed that the lesion was not present before the placement of the tattoo. We performed an excision of the nodular malignant melanoma (NMM) in June 2005 (Figure 2). A benign melanocytic nevus within the tattoo was excised, and tattoo pigment was found in the same layer of the dermis as the nevus cells (Figure 3). Subsequently, the NMM was re-excised in July 2005, with 2 cm margins combined with a sentinel-node biopsy (3/4 positive). Furthermore, a left axillary lymph node dissection revealed lymph node metastasis (9/20 positive) (Figure 4). We started a high-dose interferon therapy in March 2006, which was paused due to paresthesia in the fingertips between April and August 2006. In September 2007, multiple pronounced cerebral, cerebellar, cervical, abdominal and thoracic metastases were found. The patient was admitted to our clinic and we immediately started radiation therapy (10 sessions) and chemotherapy with Temozolomide 200 mg/kg. Due to the above-mentioned, acute, life-threatening diagnosis we needed to stop the chemotherapy and give priority to radiation therapy in the range from C7 to TH3. In addition, a gamma knife irradiation of the cerebral and cerebellar metastases was carried out. Resumption of chemotherapy with Taxol and Carboplatin was planned for November 2007, but, unfortunately, the patient died shortly after.

Sequencing was performed post mortem and did not reveal mutations in the BRAF gene.

### 3.2. Cases with the Formation of Basal Cell Carcinoma in Tattooed Skin

**Figure 5:**
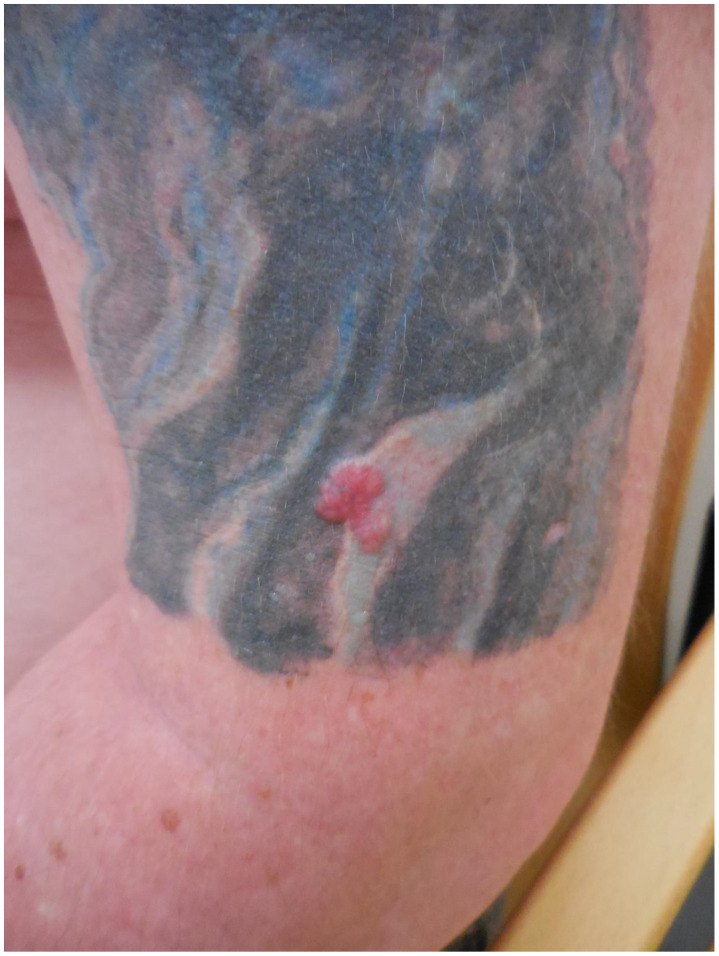
Patient with a basal cell carcinoma in tattooed skin [13].

Patient 3: A 57-year-old man visited the department of dermatology because of a 1.5-year-old lesion in his black tattoo on the right shoulder (see Figure 5). During a physical examination, we observed an erythematous glazing tumor with telangiectasia in the tattooed area. No other malignancies were found during an extensive skin examination. Anamnesis revealed the lesion was not present before placement of the tattoo. Histopathology of a skin biopsy showed a nodular basal cell carcinoma. The tumor was radically excised [13].

**Figure 6:**
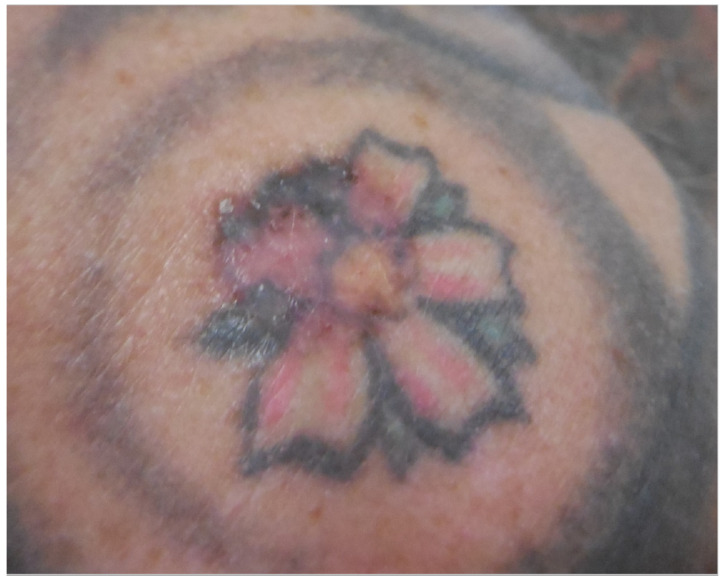
A nodular and superficial basal-cell carcinoma arising from red, black and green tattoo ink on the right shoulder. Reprinted from Ref. [19].

Patient 4: A 52-year-old man, who was otherwise healthy, was diagnosed with a basal cell carcinoma on the right shoulder in a multi-colored tattoo (see Figure 6). No other malignancies were found during an extensive skin examination. Anamnesis revealed that the lesion was not present before placement of the tattoo. The tumor was radically excised. 

**Figure 7:**
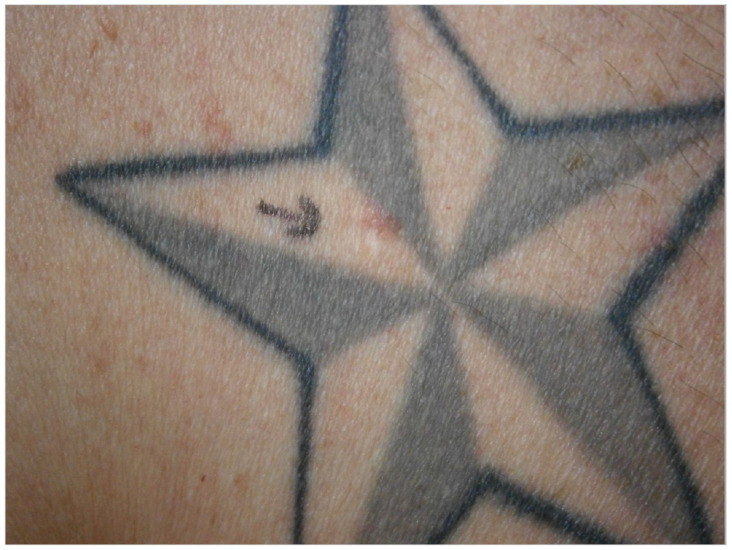
A patient with five shiny plaques on the back.

Patient 5: A 50-year-old man with a medical history of multiple basal cell carcinomas, presented with four erythematous shiny plaques, in the Department of Dermatology (see Figure 7). No other malignancies were found during an extensive skin examination. Anamnesis revealed that the lesion was not present before placement of the tattoo. The histopathology of one of the lesions confirmed the suspected superficial basal cell carcinoma. The lesions were treated with topical 5-fluorouracil 5% ointment.

**Figure 8:**
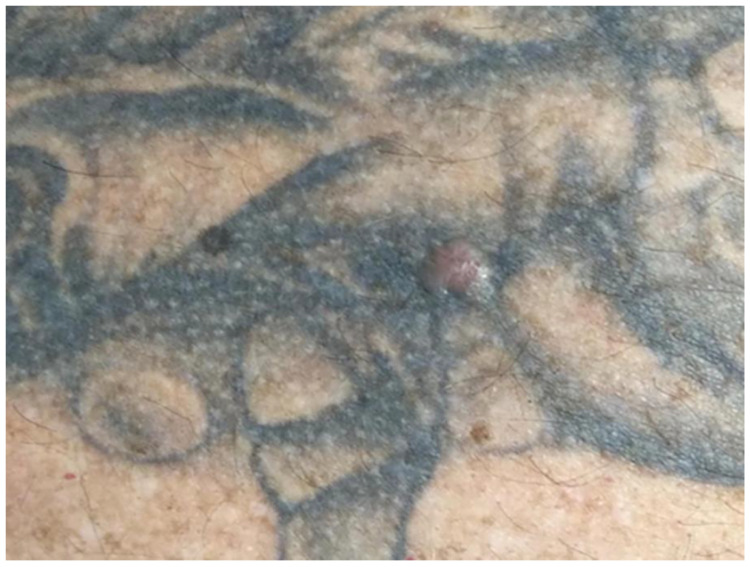
Patient 6: A patient with BCC on his back, paravertebral left side.

Patient 6: The last case is of a 60-year-old male truck driver who presented in the Department of Dermatology because of a lesion on the back (unknown time period). The patient’s medical history revealed hypertension and the diagnosis of diabetes mellitus type 2. Furthermore, he was already diagnosed previously with BCC and SCC in an area of his neck. A physical examination revealed an asymptomatic pearly nodule with linear arborizing telangiectasis on his left back side paravertebral, in the area of a black tattoo (see Figure 8). After excision, histopathological examination confirmed the diagnosis of a nodular BCC.

## 4. Discussion

Although the occurrence of carcinogenic substances in tattoo ink is proven, a directly causative relationship with cutaneous malignancies is unconfirmed. Including our cases, a total of 36 cases with malignant melanoma and 18 cases of basal cell carcinoma were reported. Other reported cutaneous malignancies included squamous cell carcinoma, keratoacanthoma, dermatofibrosarcoma protuberans and cutaneous leiomyosarcoma (see Table 1).

The different possible multifactorial causes for the development of skin cancer in a tattoo were documented [2,24]. First of all, the punctuation of the skin whilst tattooing is regarded as a massive skin trauma [2,10,14]. Furthermore, the photoallergic effects caused by the exposition of UV light to the ink ingredients, and the following chronic inflammatory reaction that occurs, could represent a possible explanation for the malignant transformation in tattoos. In addition, phototoxic effects were shown to induce hydroxyl radicals causing oxidative stress [9,10,14,24,89]. Aside from this, the ink compounds and their cleavage products, which are present during laser-assisted removal or UV light exposure, could act as a tumor promotor in tattooed skin [2,14,24]. A pyrolysis study revealed the formation of cyanide, benzene, and naphthalene as cleavage products of organic pigments, which might be comparable to the products following laser-assisted removal [90]. Other studies pointed out that the cause of tattoo-related skin reactions is not only metals (e.g., titanium, barium, aluminum and copper), which are used as colorants in older tattoo ink, but also preservatives, formulants, dispersants and impurities [81,91]. More details are presented in Table S1. Interestingly, it was revealed in the study by Henrik Hering et al. that fibroblasts absorb tattoo pigments and that the tattoo pigment TiO2 significantly decreases cell viability and increases interleukin-8 release in fibroblast monolayers [92]. 

An animal study, however, showed no signs of malignancy formation in mice that were extensively tattooed after 1 y follow up, neither in the smaller subgroup of 22 UV light-exposed animals [93]. In contrary, in another study on 99 hairless mice exposed to UV light and red tattoo ink (2-anisidine), the ink was identified as slightly cocarcinogenic when the growth rate of the second tumor increased (squamous cell carcinoma) [94].

### 4.1. Melanoma and Black Tattoo Ink; Difficulties in Defining Etiopathogenesis

In our presented cases, we report the appearance of malignant melanoma in two males with black tattoo ink. Since 1938, 36 cases with malignant melanoma have been published, from which 17 cases appeared in black tattoo ink. In our cases, the patients’ tattoos contained older ink pigments, and the aforementioned carcinogenic substances might have acted as tumor promotors. Multiple hazardous compounds in black tattoo ink such as the softener substance dibutyl phthalate, hexachloro-1,3-butadiene, methenamine, dibenzofuran, benzophenone, and 9-fluorenone were confirmed in another study [95]. Especially, genotoxic polycyclic aromatic hydrocarbons dibenzofurans and 9-fluorenone, found in black tattoo ink, could act as a tumor promotor for malignant lesions in tattoos. In addition, a study showed that black tattoo ink contained nano particles and that this tattoo ink induced the cell death of fibroblasts, even in diluted form [89]. Another study found a strong association between polychlorinated biphenyls (PCBs), a compound with similar effects such as PAHs (found in older black carbon ink), and the risk of developing cutaneous malignant melanoma. In this cited study, it is suggested that PCBs act as a tumor promoter, which might promote the transformation of melanocyte clones (nevi) [96]. Likewise a higher incidence of cutaneous malignancies has been reported in PCB exposed individuals [97].

The large amounts of tattoo ink were found in tattooed skin specimens and regional lymph nodes, even years after tattooing, represented a chronic exposure. We also found the presence of tattoo ink in a lymph node with melanoma metastasis (Figure 4) [81]. Interestingly, in one of our cases of melanoma with tattoo ink (Figure 2), we found a benign melanocytic nevus component in association with black-tattoo pigmentation (Figure 3). The hypothesis that a tattoo within a benign nevus could be a risk factor for developing a melanoma is very intriguing. It might be possible that the nevus is formed following DNA damage in the melanocyte as a result of tattoo ink. We hypothesize that carcinogenic compounds in tattoo ink act as tumor promoters, which might promote the transformation of melanocyte clones (nevi) as seen in other study on PCBs [96]. Whether a melanoma is formed depends most likely on the immunological status of the individual, as well as their genetic susceptibility to DNA damage. The presence of the ink pigment in regional lymph nodes in case of metastasis also suggests the idea of a chronic exposure to the tattoo pigment in the part of the lymphatic system with a “close” relation to neoplasia.

Another possibility is the immuno-toxic effects of benzene or PAHs on the immune cells, since a higher incidence of cutaneous malignancies is reported in immunocompromised individuals [98,99]. The median age of 45 years at first diagnosis for malignant melanoma in the published tattooed individuals is lower than the age found in the general German population (60 for females and 67 for males) [100].

However, we need to keep in mind that strong evidence suggesting a causative correlation is not confirmed and the number of published cases is small.

### 4.2. Basal Cell Carcinoma in Tattoo Ink and Toxicity of Injected Pigments

We presented four cases with the formation of basal cell carcinoma. The malignancy appeared in red, green and black tattoo ink. UV-Catabolites of the red ink color differed for the used subtype and include: UV: 4-hydroxybenzamide, 4-aminobenzamide, benzamide, 4-chloroaniline, dichloroaniline, 1,4-dichlorobenzene, methoxy-naphthol AS, 2-toluidine, 2,4,5-trichloroaniline, 2-methylformanilide, 2-methylacetanilide, 2-amino-4-nitrotoluene and 4-nitrotoluene. For green tattoo ink, no UV catabolites were identified. However, products formed during the pyrolysis of the pigment green 7 included 1,2-Benzenedicarbonitrile, benzonitrile, phthalimide [90,101,102,103]. For the color orange, one study showed that the formation of a similar cleavage product (3,3′-dichlorobenzidine) following the laser treatment of Pigment Orange 13 was cyto- and genotoxic [104].

Nevertheless, we must keep in mind that the toxicity of the pigment also depends on the amount of injected pigment. One study showed that the amount of injected pigment in tattoos is approximately 2.5 mg pigment per cm^2^, which can create an upper arm tattoo of approximately 400 cm^2^. Highly tattooed people may contain even 40 g of pigment in their body [105], which corresponds to an enormous portion of possible harmful substances injected into the dermis. After the injection, the tissue swells (edema) followed by an influx of neutrophils and macrophages, partly phagocytizing these newly injected foreign substances. Over longer periods of chronic exposure, ink particles move to the deeper dermis (reticular dermis) and leave the skin via the dermal vessels and lymphatic system, which leads to entering the bloodstream and possible systemic effects [89]. However, the amounts of harmful compounds in the composition of tattoo inks seem highly variable between brands and colors, even in pigments with the same base color [85]. One study showed that the odds ratio of early-onset basal cell carcinoma was 1.8 at the site of the tattoo with the highest odds ratio for yellow and green [106]. 

The published cases contained in Table 1 show the remarkable observation that, for melanoma, 17 cases were found in black-only tattoos and 16 cases were described in colored tattoos; while in non-melanoma skin cancer, only 14 cases appeared in black-only tattoos from a total of 59 presented cases. The putative role of colored ink in non-melanoma carcinogenesis, as opposed to black ink, might necessitate further evaluation. The slightly enhanced carcinogenic potential of red ink for epithelial cancer in mice might also suggest that the carcinogenic potential of tattoo ink might be both color and tissue specific [91].

Although it is proven that tattoo ink contains harmful compounds, a causative relation between malignancies and tattoos have is unconfirmed. In addition, it should be mentioned that tattooed individuals might demonstrate a less risk-aware sun exposure habit than non-tattooed individuals, which might be another co-factor for developing skin cancer.

Mutation signatures in tattoo-associated skin cancers should be examined to separate sun-induced pathogenetic mechanisms from non-UV-induced carcinogenic mechanisms, since the mutation rate in melanomas occurring on chronically sun-exposed skin is approximately five times higher than that on skin not subject to UV exposure [107].

Long-term, prospective, epidemiologic studies on tattooed and non-tattooed individuals should be performed in order to examine a causative relationship between (cutaneous) malignancies and tattoos. The formation of melanocytic nevi in tattoos, the first step in formation of melanoma, should be examined by using photo documentation in large study groups.

## 5. Conclusions

Epidemiologically, no correlation between tattoos and the formation of malignancies is proven. However, our case series, as well as cases found in the literature research, present malignancies (malignant melanoma and basal cell carcinoma) in tattoos at a younger age compared to the epidemiologic data of general population. Furthermore, long-term, prospective research is necessary. Tattooed areas should be inspected carefully during skin examination and all cases of malignancies or other skin transformations should be reported in databases.

## Figures and Tables

**Table 1:** Reported cases of malignant melanoma, basal cell carcinoma, squamous cell carcinoma and keratoacanthoma in tattooed skin.

Tumor-Type / Cases	Gender	Median Age	Location	Occurence	Color	Date & Reference
Malignant Melanoma 36 Cases	32 male / 3 female / 1 NA	45 years (range: 9 years–82 years, 2 NA)	Back	7 (19%)	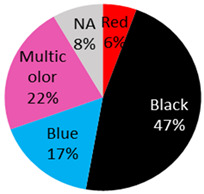	2021 (current study), 2019: [20], 2018: [2], 2016: [21], 2015: [22,23,24,25,26], 2014: [27], 2013: [5,28,29], 2011: [30] 2009: [31], 2008: [9], 2007: [32], 2006: [15,33], 2003: [34], 1999: [35], 1997: [36], 1993: [37], 1984: [10], 1980: [38], 1974: [39], 1972: [17] 1967: [40], 1938: [41]
			Shoulder	1 (3%)		
			Arm	17 (47%)		
			Leg	3 (8%)		
			Chest	3 (8%)		
			Abdomen	1 (3%)		
			Scapula	2 (6%)		
			Forehead	1 (3%)		
			NA	1 (3%)		
Basal-Cell-Carcinoma 18 Cases	13 male / 5 female	54 years (range: 28 years–76 years)	Arm	3 (17%)	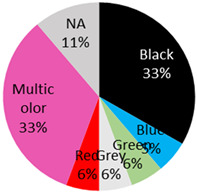	2021 (current study), 2020 [42], 2019: [43], 2018: [44], 2012: [14], 2009: [45,46], 2006: [47], 2004: [48], 1987: [49], 1983 [50]
			Back	5 (28%)		
			Eyebrow	1 (6%)		
			Hand	1 (6%)		
			Lip	2 (11%)		
			Scapula	1 (6%)		
			Shoulder	3 (17%)		
			Temple	2 (11%)		
Squamous cell carcinoma 13 Cases	5 male, 6 female, 2 NA)	47 years (range:24 years–79 years)	Arm	4 (31%)	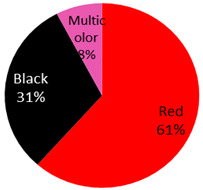	2019: [51], 2018: [52], 2017: [53,54], 2016: [55], 2015: [56], 2014: [57], 2010: [58], 2009: [59], 2008: [60], 2007: [61], 1966: [62]
			Lip	2 (15%)		
			Leg	3 (23%)		
			Back	1 (8%)		
			NA	3 (23%)		
Keratoacantoma 16 Cases	7 male, 5 female, 4 NA	54 years (range 36 years–72 years)	Scapula	1 (6%)	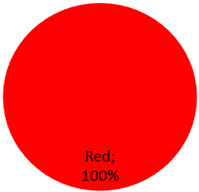	2020: [63], 2019: [64,65], 2017: [65], 2010: [66], 2009: [67], 2008: [68]
			Leg	3 (19%)		
			Arm	8 (50%)		
			Shin	2 (13%)		
			Ankle	2 (13%)		
Dermatofibrosarcoma protuberans 5 Cases	3 male, 2 female	31 years (range: 21 years–37 years, 1NA)	Arm	1 (20%)	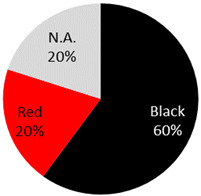	2020: [69], 2018: [70], 2014: [71], 2011: [72], 2005: [73]
			Back	2 (40%)		
			Thigh	1 (20%)		
			N.A.	1 (20%)		
Cutaneous lymphoid Hyperplasia 6 Cases	3 male, 3 female	35 years (range: 25 years–67 years)	Leg	2 (33%)	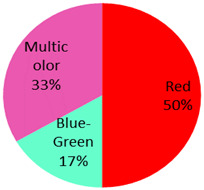	2014: [74,75], 2013: [76] 2011: [77] 2009: [78] 2002: [79]
			Shoulder	1 (40%)		
			Back	1 (17%)		
			Ankle	1 (17%)		
			N.A.	1 (17%)		
Cutaneous leiomyosarcoma 1 Case	male	41 years	Arm	1 (100%)	Black	2009: [80]

## Data Availability

No new data were created or analyzed in this study. Data sharing is not applicable to this article.

## Supplementary Materials

The following are available online at https://www.mdpi.com/article/10.3390/curroncol28060398/s1, Figure S1: PRISMA 2020 flow diagramfor literature research considering tumors in tattoos. Table S1: Tattoo colorants and its cleavage products as well as possible adverse effects.
