# Supplementary material for: Cutaneous Malignancies in Tattoos, a Case Series of Six Patients
Source: Curr Oncol. 2021 Nov 15;28(6):4721–37. doi: 10.3390/curroncol28060398 (PMC8628776; doi:10.3390/curroncol28060398)
Supplement: Supplementary file 1 [file curroncol-28-00398-s001.zip › curroncol-1335067-supplementary.pdf]

Current Oncology

MDPI

Supplementary Materials

Cutaneous Malignancies in Tattoos, a Case Series of Six Patients

Marike Leijs, Hannah Schaefer, Albert Rübben, Claudio Cacchi, Thomas Rustemeyer and Sebastiaan van der Bent

Figure S1. PRISMA 2020 flow diagram for literature research considering tumors in tattoos.

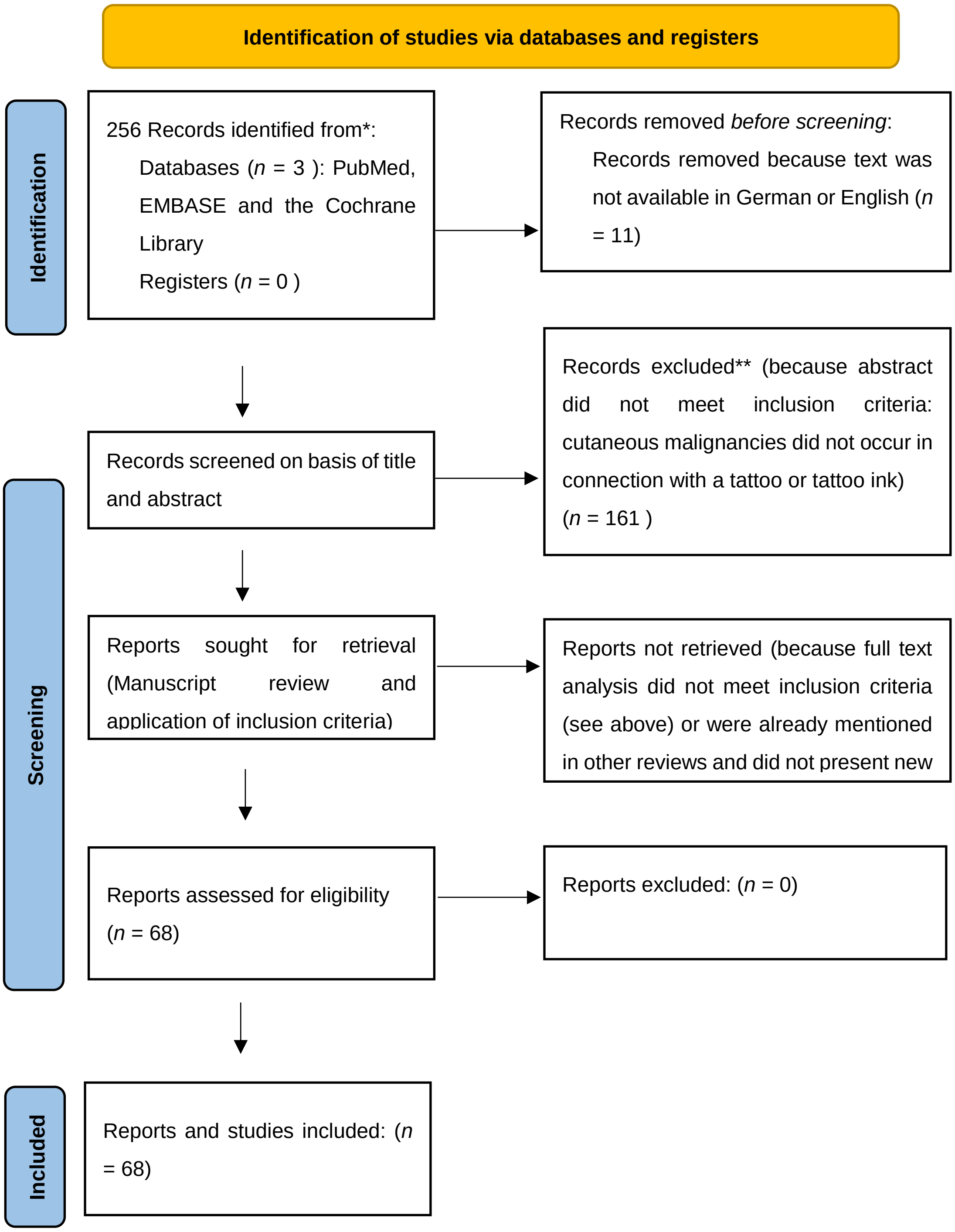

*Consider, if feasible to do so, reporting the number of records identified from each database or register searched (rather than the total number across all databases/registers). **If automation tools were used, indicate how many records were excluded by a human and how many were excluded by automation tools.

The search of Medline (PubMed, EMBASE and the Cochrane Library) provided a total of 256 results. Search Terms were (in Title or Abstract): tattoo OR ink AND melanoma (*n*=140); tattoo OR ink AND basal cell carcinoma *n*=23); tattoo OR ink AND squamous cell carcinoma (*n*=51); tattoo OR ink AND keratoacantoma (*n*=26); tattoo OR ink AND dermatofibrosarcoma protuberans (*n*=7); tattoo OR ink AND cutaneous leiomyosarcoma (*n*=1); tattoo OR ink AND cutaneous lymphoid hyperplasia (*n*=8). After adjusting for language (English or German only) 245 remained. Out of these 245 references 161 were excluded due to the fact that after reading of the abstract, the paper did not meet the inclusion criteria. The remaining 84 were then read in full text mode and examined in more detail and 18 more references were excluded because they did not fit into the searching criteria or the presented cases were already mentioned in previous reviews and no new case was introduced in the study. In the end 68 references were included in the overview in table 1.

From: Page MJ, McKenzie JE, Bossuyt PM, Boutron I, Hoffmann TC, Mulrow CD, et al. The PRISMA 2020 statement: an updated guideline for reporting systematic reviews. BMJ 2021;372:n71. doi: 10.1136/bmj.n71

For more information, visit: http://www.prisma-statement.org/, accessed on 1/9/2021

Table S1: Tattoo colorants and its cleavage products as well as possible adverse effects

Colorant	Cleavage Products (UV/Laser/Pyrolysis)	Reference
CI Pigment Yellow 74:<br>IUPAC: (2-[(2-methoxy-4-nitrophenyl)diazenyl]-*N*-(2-methoxyphenyl)-3-oxobutanamide)	3-oxobutanamide, N,N0-bis(2-methoxyphenyl)urea, o-acetoacetanisidide, 2-methoxyacetanilide<br>LASER: none<br>Toxic: skin irritation, serious eye irritation	2004: [1], 2013: [2], 2020: [3]
CI Pigment Yellow 97:<br>IUPAC: N-(4-Chlor-2,5-dimethoxyphenyl)-2-[[2,5-dimethoxy-4-[(phenylamino)sulfonyl]phenyl]azo]-3-oxobutyramid	UV: none<br>LASER: aniline	2013: [2], 2020: [3]
CI Pigment Yellow 138:<br>IUPAC: 4,5,6,7-tetrachloro-2-[2-(4,5,6,7-tetrachloro-1,3-dioxoinden-2-yl)quinolin-8-yl]isoindole-1,3-dione	Laser/Pyrolyse: hydrogen cyanide, benzene, Xylenes, 1,2,3,4-tetrachlorobenzene, pentachlorobenzene, 2,3,4,5,- tetrachlorobenzylaldehyde, hexachlorbenzene, 4,5,6,7- tetrachloro1,3-isobenzofuradione, 3,4,5,6- tetrachlorophthalimide<br>Toxic:n.a.	2016: [4]<br>[5], 2020: [3]
CI: Pigment Yellow 14<br>IUPAC: 2,2′-[(3,3′-Dichlor[1 ,1′-biphenyl]-4,4′-diyl)bis(azo)]bis[N-(2-methylphenyl)-3-oxobutyramid]	UV: 2-methylformanilide, 2-methyl-acetanilide, 3,3′-dichlorodiphenyl, 3,3′-dichlorobenzidine<br>LASER: 3,3′-dichlorobenzidine	2015/2007: [6,7], 2020: [3]
CI: Pigment Yellow 83<br>IUPAC: 2,2′-((3,3′-Dichlor(1,1′-biphenyl)-4,4′-diyl)bis(azo))bis(N-(4-chlor-2,5-dimethoxyphenyl)-3-oxobutyramid)	UV: not studied<br>LASER: 3,3′-dichlorobenzidine	2013: [2], 2020: [3]
CI: Pigment Red 22<br>IUPAC: 3-Hydroxy-4-[(2-methyl-5-nitrophenyl)azo]-N-phenylnaphthalin-2-carboxamid	UV: 2-amino-4-nitrotoluene, 4-nitrotoluene<br>LASER: 2-amino-4-nitrotoluene, 4-nitrotoluene, naphthol AS	2004:[6], 2020:[3]
CI: Pigment Red 112<br>IUPAC: 3-Hydroxy-N-(o-tolyl)-4-[(2,4,5-trichlorphenyl)azo]naphthalin-2-carboxamid	UV: 2-toluidine, 2,4,5-trichloroaniline, 2-methylformanilide, 2-methylacetanilide<br>LASER: 2-toluidine, 2,4,5-trichloroaniline<br>Toxic: low (allergic skin reactions)	2013:[2], 2011:[8], 2020:[3]
CI: Pigment Red 9<br>IUPAC: 4-[(2,5-Dichlorphenyl)azo]-3-hydroxy-N-(2-methoxyphenyl)naphthalin-2-carboxamid	UV: not studied<br>LASER: 2,5-dichloroaniline, 1,4-dichlorobenzene, methoxy-naphthol AS	2013:[2], 2011:[8], 2020:[3]
CI: Pigment Red 170<br>IUPAC: 4-[[4-(Aminocarbonyl)phenyl]azo]-N-(2-ethoxyphenyl)-3-hydroxynaphthalin-2-carboxamid	UV: 4-hydroxybenzamide, 4-aminobenzamide, benzamide<br>LASER/pyrolysis: Aniline, benzene, 2-ethoxyaniline, 4-aminobenzonitrile, 2-naphtol, 2 additional unknows substances<br>Toxic: : low (allergic skin reactions)	2013:[2], 2016 [4], 2020:[3]
CI: Pigment Red 254	UV: none identified	

IUPAC: 1,4-bis(4-chlorophenyl)-3-hydroxy-2H-pyrrolo[3,4-c]pyrrol-6-one	LASER/Pyrolysis: Benzene, Chlorobenzene, 4-chlorobenzonitrile	2013:[2], 2016 [4], 2020:[3]
CI: Pigment Red 202<br>IUPAC: 2,9-dichloro-5,12-dihydroquinolino[2,3-b]acridine-7,14-dione	UV: 4-chloroaniline<br>LASER: none identified	2013:[2], 2020:[3]
CI: Pigment Red 122<br>IUPAC: 2,9-dimethyl-5,12-dihydroquinolino[2,3-b]acridine-7,14-dione	UV: none identified<br>LASER: none identified<br>Toxic: none classified	2013:[2], 2020:[3]
CI: Pigment Orange 13<br>IUPAC: 4,4′-[(3,3′-Dichlor[1,1′-biphenyl]-4,4′-diyl)bis(azo)]bis[2,4-dihydro-5-methyl-2-phenyl-3H-pyrazol-3-on]	UV: 3,3′-dichlorodiphenyl, 3,3′-dichlorobenzidine<br>LASER/Pyrolysis: aniline, 3,3′-dichlorobenzidine, : 3,3′-di-chloro-1,1`diphenyl, Benzene, 3,3′-dichlorobenzidine, Phenzlisocyanate, 3,3′-dichlorodiphenyl-4-amine, hydrogen cyanide, benzonitrile, 2-chloroaniline, 2-aminobenzonitrile	2011:[8], 2013:[2], 2016:[4], 2018: [5], 2020:[3]
CI: Pigment Orange 34<br>IUPAC: 4,4′-[(3,3′-Dichlor[1,1′-biphenyl]-4,4′-diyl)bis(azo)]bis[2,4-dihydro-5-methyl-2-(p-tolyl)-3H-pyrazol-3-on]	UV: 3,3′-dichlorobenzidine<br>LASER: not studied	2013:[2], 2020:[3]
CI: Pigment Orange 16<br>IUPAC: 2,2′-[(3,3′-Dimethoxy[1,1′-biphenyl]-4,4′-diyl)bis(azo)]bis[3-oxo-N-phenylbutyramid]	UV: formanilide, acetanilide, 3,3′-dimethoxydiphenyl<br>LASER: none identified	2013:[2]<br>2011:[8], 2020:[3]
CI: Pigment Violet 23<br>IUPAC: 2,20-Dichloro-13,31-diethyl-18,36-dioxa-4,13,22,31-tetraza-nonacyclo (19.15.0.03,19.05,17.06,14.07,12.023,35.024,32.025,30) hex-atriaconta-1,3,5(17),6(14),7,9,11,15,19,21,23(35),24(32),25,27,29,33-hexadecaene	UV: none identified , LASER: none identified, Pyrolysis: Benzene, 2-amino-9-oxo-dihydroacridine-3-carbaldehyde,	2013:[2], 2020:[3]
CI: Pigment Violet 19<br>IUPAC : 5,12-dihydroquinolino[2,3-b]acridine-7,14-dione	Pyrolysis: 1,2-Benzenedicarbonitrile, benzonitrile, phthalimide, UV: none identified, LASER: none identified, Component toxicity (Copper): acute oral toxicity, serious eye irritation, acute inhalation toxicity	2016: [4], 2020:[3]
CI: Pigment Green 7<br>IUPAC: Polychloro copper phthalocyanine	UV: none identified<br>LASER/Pyrolysis: 1,2-benzene dicarbonitrile, 2-methylben-zonitrile, benzonitrile, benzene, and the poisonous gas hy-drogen cyanide (HCN)	2013:[2]
CI: Pigment Blue 15<br>IUPAC: (29H,31H-phthalocyaninato(2-)-N29,N30,N31,N32)copper	UV:n.a., Laser:n.a.<br>Toxic: may cause respiratory irritation (Specific target organ toxicity, single exposure), suspected of causing cancer (Car-cinogenicity)	2016: [4], 2020:[3]
CI: Pigment Black 7<br>IUPAC: Carbon Black	Toxic: Chronic (Cumulative) Toxicity, Toxic to Reproduc-tion (ECHA) suspected, Tumorigenic, Carcinogenic, irrita-tive	2013:[2], 2016: [4], 2020:[3]
**Contamination/additives**		
CI/IUPAC: Antimony	Tumorigenic, Carcinogenic, irritative	2020:[3]
CI/IUPAC: Arsenic	Toxic: acute & chronic toxic, Carcinogenic, irritative	2020:[3]
CI/IUPAC: Cadmium	Toxic: Carcinogenic (inhalative), toxic, irritative	2020:[3]
CI/IUPAC: Chromium	Toxic: Irritative, anticipated to be human carcinogen, acute toxic, cumulative lung damage	2020:[3]
CI/IUPAC: Cobalt	Toxic: probable human carcinogen, nervous system disturb-ance, acute toxic, irritative	2020:[3]
CI/IUPAC: Lead	Toxic: anticipated to be human carcinogen, irritative, chronic (cumulative) toxic, mutagen	2020:[3]
CI/IUPAC: Nickel	Toxic: Skin sensitizer (Category 1), irritative (eyes, nose, throat, skin)	2020:[3]
CI: Methyl methacrylate<br>IUPAC: methyl 2-methylprop-2-enoate	Toxic: Skin sensitizer (Category 1), irritative (eyes, nose, throat, skin)	2020:[3]
CI: Dodecyl methacrylate<br>IUPAC: dodecyl 2-methylprop-2-enoate	Toxic: Skin sensitizer (Category 2), serious irritative (eyes, nose, throat, skin), may cause respiratory irritation	2020:[3]
CI: Tetradecyl methacrylate<br>IUPAC: tetradecyl 2-methylprop-2-enoate	Toxic: Skin irritation, may cause allergic skin reaction, seri-ous eye irritation, may cause respiratory irritation	2020:[3]
IUPAC: Styrene	Toxic: Skin sensitizer (Category 2), serious irritative (eyes, nose, throat, skin), acute inhalative toxicity, specific target organ toxicity; repeated exposure (Cat. 1), suspected of re-productive toxicity	2020:[3]
CI: α-Methylstyrene<br>IUPAC: prop-1-en-2-ylbenzene	Toxic: Causes serious eye irritation, may cause respiratory irritation	2020:[3]

CI/IUPAC: Benzene	Toxic: Carcinogenicity (Cat 1a), Skin irritation, serious eye irritation, may cause genetic effects, specific organ toxicity (Repeated exposure)	2016:[4], 2020:[3]
CI/IUPAC: Toluene	Toxic: Skin irritation (Cat.2), Specific target organ toxicity, repeated exposure, Cat. 1, reproductive toxicity	2016:[4], 2020:[3]
CI/IUPAC: Naphthalene	Toxic: Carcinogenicity (Cat. 2), acute oral toxicity	2016:[4], 2020:[3]
CI/IUPAC: Biphenyl	Toxic: Skin irritation (Cat.2), serious eye irritation, may cause respiratory irritation	2016:[4], 2020:[3]
CI: Dimethylcyclosiloxanes (oligomers)<br>IUPAC: 2,2-dimethyl-1,3,5,2,4,6-trioxatrisilinane	Toxic: Reproductive toxicity, Cat. 2	2016:[4], 2020:[3]
CI/IUPAC: Silicone	Toxic: Serious eye irritation (Cat. 2b)	2016:[4], 2020:[3]
CI: Ethylene glycol<br>IUPAC: ethane-1,2-diol	Toxic: acute oral toxicity, Specific target organ toxicity, repeated exposure (kidney), Cat. 2	2016:[4], 2020:[3]
CI: 1-Methyl-2-pyrrolidinone<br>IUPAC: 1-methylpyrrolidin-2-one	Toxic: Skin irritation (Cat. 2), serious eye irritation, Specific target organ toxicity, (single exposure; Respiratory tract irritation), Reproductive toxicity(teratogenic effects)	2016:[4], 2020:[3]
CI: N-Vinylpyrrolidone<br>IUPAC: 1-ethenylpyrrolidin-2-one	Toxic: Carcinogenicity (Cat. 2), acute oral toxicity, acute dermal toxicity, serious eye irritation, acute inhalation toxicity, may cause respiratory irritation (Specific target organ toxicity, single exposure; Respiratory tract irritation)	2016:[4], 2020:[3]

References

1. Cui, Y.; Spann, A.P.; Couch, L.H.; Gopee, N.V.; Evans, F.E.; Churchwell, M.I.; Williams, L.D.; Doerge, D.R.; Howard, P.C. Photodecomposition of Pigment Yellow 74, a Pigment Used in Tattoo Inks. *Photochem. Photobiol.* **2004**, *80*, 175–184.
2. Wezel, K. Untersuchung des Verhaltens von Tätowiertinten und Pigmenten unter Lichteinfluss. Thesis, University of Giessen, Giessen, Germany, 2013.
3. PubCHEM. National Center for Biotechnology Information. PubChem Database. Carbon, CID=5462310, PubCHEM2020. Available online: http://URL (accessed on 1 February 2021)
4. Schreiver, I.; Hutzler, C.; Andree, S.; Laux, P.; Luch, A. Identification and hazard prediction of tattoo pigments by means of pyrolysis—gas chromatography/mass spectrometry. *Arch. Toxicol.* **2016**, *4*, 1639–1650.
5. Hering, H.; Sung, A.Y.; Röder, N.; Hutzler, C.; Berlien, H.-P.; Laux, P.; Luch, A.; Schreiver, I. Laser Irradiation of Organic Tattoo Pigments Releases Carcinogens with 3,3′-Dichlorobenzidine Inducing DNA Strand Breaks in Human Skin Cells. *J. Investig. Dermatol.* **2018**, *138*, 2687–2690.
6. Vasold, R.; Naarmann, N.; Ulrich, H.; Fischer, D.; Konig, B.; Landthaler, M.; Bäumler, W. Tattoo pigments are cleaved by laser light-the chemical analysis in vitro provide evidence for hazardous compounds. *Photochem. Photobiol.* **2004**, *80*, 185–190.
7. Engel, E.; Spannberger, A.; Vasold, R.; König, B.; Landthaler, M.; Bäumler, W. Photochemical cleavage of a tattoo pigment by UVB radiation or natural sunlight. *J. Dtsch. Dermatol. Ges.* **2007**, *5*, 583–589.
8. Gaugler, S. Analysis of bioactive compounds in tattoo inks before and after irradiation with sunlight using HPTLC and in situ detection with vibrio fischeri. Thesis, University of Hohenheim, Stuttgart, Germany, 2011.
9. Schreiver, I.; Hutzler, C.; Laux, P.; Berlien, H.-P.; Luch, A. Formation of highly toxic hydrogen cyanide upon ruby laser irradiation of the tattoo pigment phthalocyanine blue. *Sci. Rep.* **2015**, *5*, 12915.
